# Oral Galvanism Side Effects: Comparing Alloy Ions and Galvanic Current Effects on the Mucosa-like Model

**DOI:** 10.3390/jfb14120564

**Published:** 2023-12-11

**Authors:** Natalia Chepelova, Artem Antoshin, Sergei Voloshin, Anna Usanova, Yuri Efremov, Maria Makeeva, Stanislav Evlashin, Mikhail Stepanov, Anna Turkina, Peter Timashev

**Affiliations:** 1 Institute for Regenerative Medicine, Sechenov First Moscow State Medical University, 8-2 Trubetskaya St., Moscow 119048, Russia; chepelova_n_k@staff.sechenov.ru (N.C.); voloshin_s_yu@staff.sechenov.ru (S.V.); usanova_a_p@staff.sechenov.ru (A.U.); efremov_yu_m@staff.sechenov.ru (Y.E.); timashev_p_s@staff.sechenov.ru (P.T.); 2 Therapeutic Dentistry Department, Institute for Dentistry, Sechenov First Moscow State Medical University, 8-2 Trubetskaya Str., Moscow 119048, Russia; makeeva_m_k@staff.sechenov.ru (M.M.); turkina_a_yu@staff.sechenov.ru (A.T.); 3 Conservative Dentistry Department, RUDN University, 6 Miklukho-Maklaya Street, Moscow 117198, Russia; 4 Center for Materials Technologies, Skolkovo Institute of Science and Technology, Moscow 121205, Russia; stevlashin@gmail.com; 5 Department of Dental Surgery, Sechenov First Moscow State Medical University, 8-2 Trubetskaya Str., Moscow 119048, Russia; stepanov_m_a@staff.sechenov.ru

**Keywords:** dental alloys, biocompatibility, toxicity, side effects, galvanic corrosion, galvanic current, hypersensitivity, galvanism, tribocorrosion, cell model

## Abstract

The interaction of different dental alloys with the oral environment may cause severe side effects (e.g., burning sensation, inflammatory reactions, carcinogenesis) as a result of oral galvanism. However, the pathogenesis of side effects associated with oral galvanism is still unclear, and the effects of direct current and alloy corrosion ions are considered potentially contributing factors. Therefore, the aim of this study was to systemically compare the damaging effects of (1) galvanism as a synergistic process (direct current + corrosion ions), (2) direct current separately, and (3) corrosion ions separately on an in vitro mucosa-like model based on a cell line of immortalized human keratinocytes (HaCaTs) to reveal the factors playing a pivotal role in dental alloys side effects. For this, we chose and compared the dental alloys with the highest risk of oral galvanism: Ti64–AgPd and NiCr–AgPd. We showed that galvanic current may be the leading damaging factor in the cytotoxic processes associated with galvanic coupling of metallic intraoral appliances in the oral cavity, especially in the short-term period (28 days). However, the contribution of corrosion ions (Ni^2+^) to the synergistic toxicity was also shown, and quite possibly, in the long term, it could be no less dangerous.

## 1. Introduction

Metallic oral appliances are widely used in dentistry. The most common alloys are as follows: commercially pure titanium, Ti6Al4V, cobalt–chromium, nickel–chromium, and nickel–titanium [1]. Nevertheless, the interaction of different dental alloys with the oral environment may cause local and systemic side effects in the human body [2]. The intraoral manifestations may vary from a burning sensation and local inflammatory reaction to the development of potentially malignant oral disorders, such as lichenoid reaction and oral verrucous leukoplakia [3,4,5,6].

However, the pathogenesis of side effects associated with dental alloys is still unclear. The following three mechanisms are supposed to cause tissue damage: (1) cytotoxicity of metal ions [7], (2) allergic reaction to metal ions [8], and (3) effect of galvanic current occurring between different metallic appliances [3]. Galvanic current occurs between metallic appliances produced from different alloys via saliva, which serves as an electrolytic medium [9]. The strength of galvanic current depends on the potential difference between the alloys and on the saliva composition [2]. The highest value of direct current (up to 102 µA) has been observed in galvanic couples of dental amalgam with other dental alloys [10]. The current density in galvanically coupled titanium implants/suprastructures in artificial saliva may vary from 0.5 up to 12 µA/cm^2^ depending on the suprastructure alloy [11]. On the one hand, the galvanic current increases corrosion and ion release from metallic appliances [12], and on the other hand, it itself may have a damaging effect on oral tissues [3]. These two mechanisms act synergistically in the oral cavity, and it is difficult to assess their effects separately.

The biocompatibility of different dental alloys has been well studied. In vitro assessment of dental alloys cytotoxicity is standardized by ISO 7405:2018 [13]. It was shown that metal ions may damage epithelial cells and periodontal fibroblasts [14,15], which may lead to chronic inflammation [7]. Moreover, oxidative stress caused by metal ions may increase the risk of potentially malignant oral disorders [16,17]. Ni^2+^ is supposed to be the most cytotoxic ion released from dental alloys [18]. Conversely, Ti was shown to be the most biocompatible element; however, it may also cause tissue inflammation or damage in case of increased corrosion of dental implants [19,20].

On the other side, the effect of galvanic current on the oral mucosa is ambiguous and under-researched. Wartenberg et al. registered the apoptosis of oral mucosa cancer cells in response to direct current (DC) electrical fields [21]. Podzimek et al. applied DC on human lymphocytes, which resulted in a decreased proliferation and altered surface marker expression at critical values above 5 µA [22]. Korraah et al. investigated the effect of DC electric fields on leukoplakia cells and concluded that chronic galvanic current might cause malignization of oral lesions [23]. However, these studies were conducted using different cell cultures and at different current values. Of note, in contrast, in recent years, it has been shown that direct currents may be used for electrical stimulation of wound healing due to the electrotaxis process [24,25].

Thus, studies on the effects of potentially cytotoxic factors produced as a result of oral galvanism (metal ions and current) are rather sporadic, and moreover, they have been performed on different cell types. In addition, in the case of the direct current, its effect and consequences on mucosal cells, particularly keratinocytes, are not fully understood. To the best of our knowledge, there is no comprehensive study that has examined the effects of oral galvanism in a single experiment on a standardized model of oral mucosa.

Therefore, the aim of this study was to systemically compare the damaging effects of (1) galvanism as a synergistic process, (2) direct (constant) current separately, and (3) corrosion ions separately on an in vitro mucosa-like model based on a standardized cell line of immortalized human keratinocytes (HaCaTs) to reveal the factors playing a pivotal role in dental alloys side effects. Taking into account the increasing number of people having metallic intraoral appliances [26], this study seems to have substantial clinical significance.

## 2. Materials and Methods

### 2.1. Galvanic Couple Testing

The samples for galvanic coupling were prepared from different dental alloys used in prosthetic dentistry. The cobalt–chromium alloy (Co–Cr) [Techkom, Ulyanovsk, Russia]: Co 65%, Cr 28%; Mo 5%, others 2%; silver–palladium dental casting alloy (Supermetall, Moscow, Russia): Ag 74%, Pd 25%, others 1%; Ti64 dental alloy for metal–ceramic (TitanMetService, St. Petersburg, Russia): Ti 90%, Al 6%, V 5%; NiCr dental alloy for metal–ceramic (Techkom, Ulyanovsk, Russia): Ni 63%, Cr 25%, Mo 10%, others 2%. All stated percentages are considered as wt.%. The samples were in the form of cylinders with a diameter of 10 mm and a length of 20 mm. Samples were used as received without surface treatment.

To measure the potential differences between different couples of alloys, the surfaces of samples were processed according to the protocol used in the manufacturing of prosthetic intraoral appliances [27]. The galvanic cell for the experiment was custom manufactured using 3D printing, the distance between the electrodes (different dental alloys) was 8 mm, and the depth of immersion in the electrolyte was 10 mm. The following alloy combinations were tested: Ti64–AgPd, NiCr–AgPd, CoCr–AgPd, CoCr–Ti64, NiCr–Ti64, CoCr–NiCr.

Fuzayama–Meyer’s artificial saliva was used as a medium (pH = 6.50, electrical conductivity 3540 ± 120 μS/cm). The solution was prepared using 0.4 g of NaCl, 0.4 g of KCl, 0.6 g of CaCl_2_, 0.58 g of Na_2_HPO_4_, and 1 g of CH_4_N_2_O solubilized in distilled water to achieve a total volume of one liter. The open circuit potential between different metal pairs was measured for 2 h using a Potentiostat/Galvanostat Elins PX40 (Electrochemical Instruments, Chernogolovka, Russia). Open circuit potential was measured for 3 pairs of samples, and the average values were taken. For further experiments, the alloys with maximum potential differences (Ti64–AgPd and NiCr–AgPd) were used.

### 2.2. Artificial Saliva Saturation with Corrosion Ions

Both alloy samples and artificial saliva were preliminarily autoclaved for 30 min at 121 °C to ensure sterility. For saturating the artificial saliva with corrosion ions, specimens of NiCr, Ti64, and AgPd alloys were chosen, and they were coupled as follows: Ti64–AgPd (named as Ti64–AgPd-i group), NiCr–AgPd (named as NiCr–AgPd-i group).

According to ISO 7405:2018 standard [13], these pairs were placed into Eppendorf tubes filled with artificial saliva with sample surface area to solution ratio of 3.0 cm^2^/mL [28]. Subsequently, the tubes were kept in a CO_2_ incubator with 5% CO_2_ and 37 °C for 72 h. This procedure was repeated multiple times until a volume of the saturated artificial saliva was enough to carry out subsequent experiments.

To measure the electric conductivity of the saturated artificial saliva, impedance spectroscopy operating within a frequency spectrum ranging from 1 to 500 kHz and an alternating current signal amplitude of 30 mV was employed. The same Potentiostat/Galvanostat Elins PX40 was used for the investigation. All measurements were performed at ambient temperature (23 °C) utilizing a reversible hydrogen electrode. Corrosion assays were executed within a potential span of −1 to 5 V, with the upper threshold of potential established at 5 V. The scanning velocity was set at 10 mV s^−1^ for sample measurement. All measurements were carried out for three independent samples, after which the average value was taken.

### 2.3. Fabrication of Oral Mucosa-like Model

A membrane composed of type I collagen was used as a substrate for cell seeding in the fabrication of the mucosa-like model. Briefly, type I collagen was extracted from bovine tendons in a fibrillar form, and subsequently, it was electrophoretically deposited with the assistance of semipermeable barrier according to the technique that we previously described [29]. The obtained collagen membranes (1 × 1 cm) were lyophilized for 24 h at −40 °C to obtain porosity.

The HaCaT cell line, which is immortalized adult human skin keratinocytes, was taken from Sechenov University Biobank for the development of an in vitro model of oral mucosa-like [30]. The cells were cultured using growth medium of the following composition: DMEM/F12 (1:1, BioloT, St. Petersburg, Russia), gentamicin (50 μg/mL, PanEco, Moscow, Russia), fetal calf serum FBS (10%, Thermo Fisher Scientific, Waltham, MA, USA). The cells were seeded onto one (upper) surface of the collagen membrane at a density of 250,000 cells per membrane (1 cm^2^) [31] and cultured for three days at 37 °C. Subsequently, the cell-laden collagen membranes were positioned on custom-made 3D-printed constructs designed for cell cultivation at the air–liquid interface (ALI), and thus, the oral mucosa-like model was established. These constructs (*n* = 18) were then installed into the wells of the 12-well plates. Cell growth medium was poured into wells to moisturize the underside of the collagen membrane, while the upper surface with cells was in contact with air.

### 2.4. Exposition of Mucosa-like Model

A total of 6 groups were established in this experiment (*n* = 3 mucosa-like models per each). The samples of different alloys in the form of 8 mm long and 2 mm thick rods were positioned onto the opposite sides of mucosa-like model so that the distance between them was 6 mm. The following alloy couples (groups) were organized on the ALI: (1) Ti64–AgPd and (2) NiCr–AgPd. Similarly, the gold rods connected to DC power supply were placed onto ALI, and the constant current of 10 µA was generated ((3) current group). The ALI with rods was moisturized with 100 µL of artificial saliva solution 2 times a day.

The air–liquid interface was also treated with 100 µL of artificial saliva solution saturated with corrosion ions twice a day: (4) Ti64–AgPd-i and (5) NiCr–AgPd-i groups, respectively. As well, pure artificial saliva ALI treatment (100 µL/twice a day) was performed as a control group ((6) artificial saliva).

The well plates containing the assembled mucosa-like models were placed into CO_2_ incubator with 5% CO_2_ at 37 °C for 28 days. The cell growth medium in the wells was refreshed every 2 days.

### 2.5. Toxicity Evaluation

#### 2.5.1. Metabolic Activity and Proliferation

Following a 28-day experimental period, the metabolic activity of the mucosa-like models was measured by the AlamarBlue assay (*n* = 3 for each group, five repetitions per measurement) based on resazurin reduction (Invitrogen, Waltham, MA, USA) according to the manufacturer instructions using Victor Nivo spectrofluorometer (PerkinElmer, Waltham, MA, USA) with an excitation wavelength of 530 nm and an emission wavelength of 590 nm. Subsequently, the DNA of the samples (*n* = 3 for each group, five repetitions per measurement) was quantified to measure cell proliferative activity. For this, the Quant-iT PicoGreen kit (Invitrogen, Waltham, MA, USA) was applied according to the manufacturer’s instructions using a Victor Nivo spectrofluorometer with an excitation wavelength of 480 nm and an emission wavelength of 520 nm. Additionally, the metabolic activity of cells was then normalized to the amount of DNA and expressed in relative units (U) to make comparisons among different groups valid.

#### 2.5.2. Live/Dead Assay

Cell viability on the membranes (*n* = 3 for each group) was visualized by live/dead staining. Live cells were stained with 0.5 mg/mL of calcein (Sigma-Aldrich, Waltham, MA, USA), dead cells were stained with 1.5 μM of propidium iodide (PI) (Thermo Fisher Scientific, Waltham, MA, USA), while cell nuclei were stained with 0.004 mg/mL Hoechst (Thermo Fisher Scientific, Waltham, MA, USA). The stained samples were analyzed with EVOS M5000 imaging system (Thermo Fisher Scientific, Waltham, MA, USA).

#### 2.5.3. Enzyme-Linked Immunosorbent Assay

On day 28 of the experiment, the growth medium samples were collected from the wells. The aliquots of each medium sample were subjected to enzyme-linked immunosorbent assay (ELISA) for interleukin-6 and caspase-3 detection (five aliquots per group). For this, the Human IL6 ELISA kit (Arigobio, Hsinchu City, Taiwan) and the Human Caspase-3 ELISA kit (Arigobio, Hsinchu City, Taiwan) were used, respectively, according to the manufacturer’s instructions. The optical density measurements of the solutions were performed using a Victor Nivo spectrofluorometer (PerkinElmer, Waltham, MA, USA) at a wavelength of 450 nm. Additionally, the levels of IL-6 and caspase-3 were then normalized to the amount of DNA and expressed in relative units (U).

### 2.6. Histology

The samples of mucosa-like model (*n* = 3 for each group) were fixed in 4% neutral buffered formalin overnight at 4 °C, treated with 30% sucrose solution overnight at 4 °C, and immersed in the OCT medium. Cryosections 16 μm thick were produced utilizing an Epredia Microm HM525 NX Cryostat (Carl Zeiss, Jena, Germany), followed by hematoxylin and eosin (H&E) staining. The specimens were studied utilizing a Leica DM1000 LED microscope (Leica Microsystems, Wetzlar, Germany).

### 2.7. Mechanics

The AFM measurements were performed with a Bruker Bioscope Resolve AFM (Bruker, Camarillo, CA, USA) integrated with an Axio Observer inverted optical microscope (Carl Zeiss, Jena, Germany) according to a previously described protocol [32]. Precalibrated cantilever PeakForce QNM-Live Cell probes (PFQNM-LC-A-CAL, Bruker AFM Probes, Camarillo, CA, USA) with a spring constant of 0.1 N/m were used, and the deflection sensitivity (nm/V) was calibrated by the thermal tune method. The force–volume maps were acquired over a 50 × 50 µm, 20 × 20 points region, with 5–6 regions per sample (*n* = 3 for each group). The individual force curves of the map were acquired at a 30.5 Hz ramp rate and a 3 µm vertical ramp distance, and the force set-point was ~2 nN. The approach (extend) part of the curve was processed using the previously developed MATLAB code [33]. The Hertz’s model was used to obtain the Young’s modulus (YM) *E*:

(1) $$F=\frac{4}{3}\frac{E}{1-\nu^{2}}\delta^{3/2}\sqrt{R_{}}$$

where *F* is the measured force, *δ* is the indentation depth, *R* is the tip radius, and *ν* is the Poisson’s ratio of the sample (was considered 0.5). No finite thickness correction was applied since the surface under the cells had a comparable stiffness.

The microindentation measurements were performed using a MicroTester G2 micro-scale mechanical test system (CellScale, Waterloo, ON, Canada) in the displacement control mode. Wet samples were placed on the hard sample stage. The used indenter probes had a stiffness of 0.3145 N/m and a spherical tip with a radius of 500 μm. Indentations were performed up to the depth of 200 μm. Five indentation measurements per sample (*n* = 3 for each group) were performed at different points. The load–displacement curves were processed the same way as the AFM data with the same previously developed MATLAB code [33]. The loading part of a curve was processed with Hertz’s model to obtain the YM, and a finite thickness correction was applied since the indentation depth was comparable with the sample thickness (~1 mm).

### 2.8. Statistical Analysis

The statistical analysis was performed with GraphPad Prism 9 software (GraphPad Software Inc., La Jolla, CA, USA). The Shapiro–Wilk test was performed to test the normality of data distribution. In the case of normal data distribution, a comparison by two-way analysis of variance (two-way ANOVA) followed by multiple comparisons using Tukey’s test was performed. In these cases, the results of statistical analysis are represented as means ± standard deviations. In the case of non-normal distribution, the comparison was performed using the nonparametric Kruskal–Wallis test, followed by multiple comparisons using Dunn’s test. The results are presented as median values with interquartile ranges. The differences were considered significant at *p* < 0.05.

## 3. Results

### 3.1. Galvanic Couples

**Figure 1:**
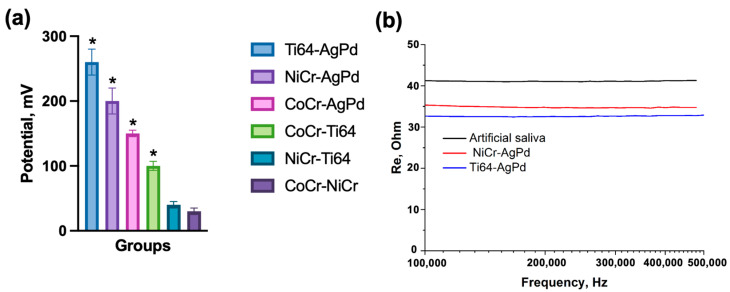
Comparison of different galvanic couples. (**a**) Measurement of potentials between different dental alloys. The data are represented as mean ± SD. (**b**) Measurement of artificial saliva solution resistance after 28-day exposition to Ti64–AgPd or NiCr–AgPd galvanic couples. * *p* < 0.05 compared with the group with less potential.

Before choosing the galvanic couples for the current experiment, we compared the most common metal alloys used in prosthetic dentistry in different combinations (Figure 1a). After potential stabilization, the following results were obtained: Ti64–AgPd—260 ± 20 mV, NiCr–AgPd—200 ± 20 mV, CoCr–AgPd—150 ± 5 mV, CoCr–Ti64—100 ± 7 mV, NiCr–Ti64—40 ± 5 mV, CoCr–NiCr—30 ± 5 mV. From these data, the couples with maximum potential were chosen for further experiments (Ti64–AgPd and NiCr–AgPd).

Subsequently, the pairs were placed into the artificial saliva (AS) solution for 28 days to initiate the corrosion processes. The results of the saliva solution resistance measurement are represented in Figure 1b. The resistance of solutions with both galvanic pairs was lower than that of the control group (artificial saliva): Ti64–AgPd ~ 32–33 Ohm, NiCr–AgPd ~ 35–36 Ohm, AS ~ 41–42 Ohm.

### 3.2. Mucosa-like Model Exposition

**Figure 2:**
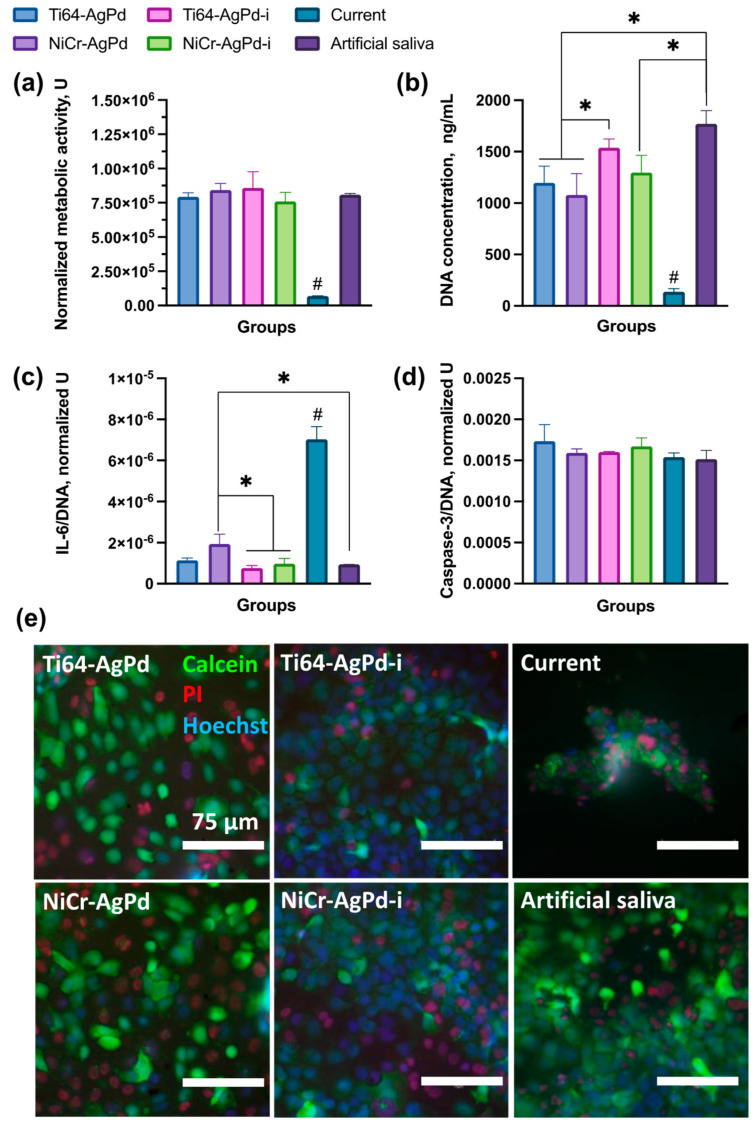
Oral mucosa-like model after exposition to different factors for 28 days: (**a**) normalized metabolic activity of cells; (**b**) proliferation of cells according to DNA levels; (**c**) levels of IL-6 normalized to DNA quantity; (**d**) levels of caspase-3 normalized to DNA quantity; (**e**) live/dead staining of cells on the surface of mucosa-like model. The data are represented as mean ± SD. * *p* < 0.05, # *p* < 0.05 compared with other groups.

The mucosa-like model was exposed to six factors: (1,2) galvanic couples (Ti64–AgPd and NiCr–AgPd) placed onto its surface, (3,4) their ions collected in the preparatory phase (Ti64–AgPd-i and NiCr–AgPd-i), (5) the constant current of 10 µA generated by the DC supply, (6) and the artificial saliva as a control group (Figure 2).

When analyzing the metabolic activity of HaCaTs as a cellular component of the mucosa-like model, it was shown that the impact of all factors was negligible except the current of 10 µA. In this case, the normalized metabolic activity of cells (6.8 ± 0.2 × 10^4^ U) dropped by at least 10 times compared with other groups (Figure 2a).

The proliferation levels in the groups had more differences. Similarly, the lowest value of proliferation according to the DNA concentration measurement (Figure 2b) was in the current group (137 ± 31 ng/mL). Expectedly, the highest level of proliferation was in the artificial saliva (control) group (1770 ± 130 ng/mL). The artificial saliva group and Ti64–AgPd-i (1539 ± 85 ng/mL) had both statistically higher proliferation than in the galvanic couples groups (1197 ± 163 ng/mL and 1079 ± 207 ng/mL for Ti64–AgPd and NiCr–AgPd, respectively). The proliferation in the NiCr–AgPd-i group (1297 ± 167 ng/mL) also decreased significantly compared with the control group. Of note, in both the galvanic couples and the ion groups, the NiCr alloy impact caused a slightly lower proliferation of cells (not statistically significant) in comparison to Ti64 alloy.

The normalized levels of proinflammatory cytokine interleukin-6 (IL-6) were the highest in the current group (7 ± 0.62 × 10^−6^ U) compared with all other groups (Figure 2c). As well, the NiCr–AgPd galvanic couple group had higher IL-6 levels (1.9 ± 0.46 × 10^−6^ U) than in both groups of ions (7.7 ± 1.22 × 10^−7^ U and 9.8 ± 2.47 × 10^−7^ U for Ti64–AgPd and NiCr–AgPd, respectively) and in the artificial saliva group (9.4 ± 0.02 × 10^−7^ U). Again, in both the galvanic couples and the ion groups, the NiCr alloy impact caused slightly higher IL-6 secretion (not statistically significant) than in the Ti64 group. Of note, the normalized levels of caspase-3 were the same for all compared groups, and no statistical differences were found (Figure 2d).

The live/dead staining (Figure 2e) of the compared groups also revealed some differences. In all the groups, there were live cells stained green, but visually, the groups of both galvanic pairs had less cell density compared with the groups of ions and artificial saliva. Nevertheless, in these groups, the cells were distributed relatively homogeneously on the whole surface of the collagen membrane. The current group had another cell distribution: the cells on the collagen membrane were aggregated into small islands sporadically located in different parts of the collagen membrane. All the groups had dead cells stained red, but in the case of the control group (artificial saliva), their number was slightly lower.

### 3.3. Histology and Mechanics

**Figure 3:**
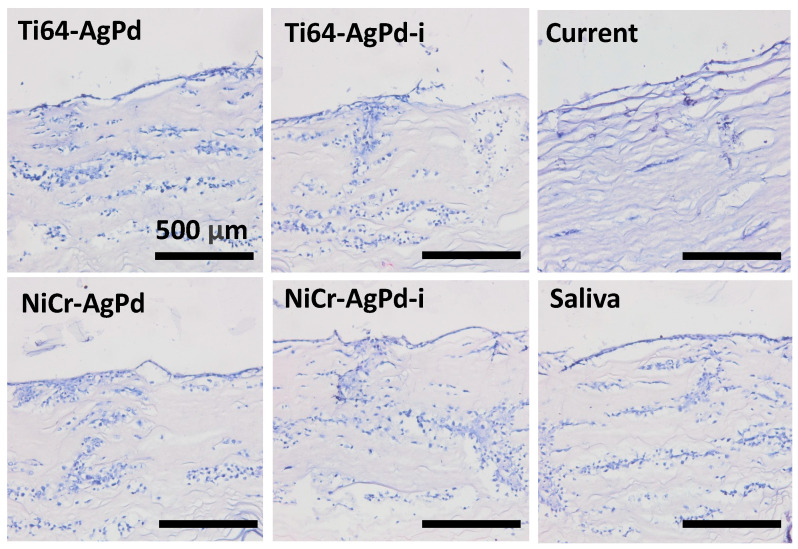
Histological analysis of mucosa-like model after exposition to different factors for 28 days. H&E staining.

Morphological evaluation of the oral mucosa-like model samples (Figure 3) showed cell spreading not only on the surface of the collagen membranes but also their migration into the porous membrane thickness in all investigated groups. However, in the case of the current group, the migration was weaker, and the cells were only in the superficial membrane layers. Moreover, their number and density were lower compared with other groups.

**Figure 4:**
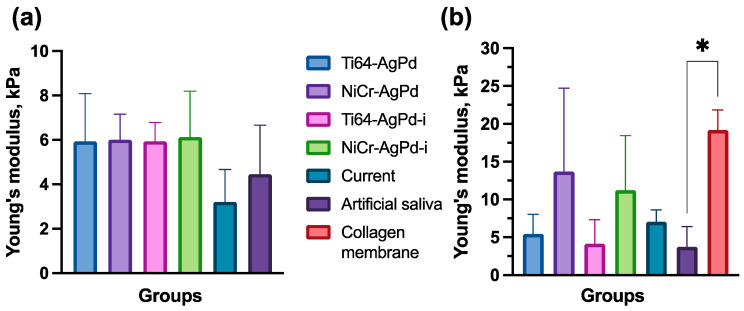
Mechanical properties of mucosa-like model. The mechanical properties of the cells were analyzed by AFM (**a**) and of the whole mucosa-like model by microindentation (**b**). The data are represented as medians and interquartile ranges. * *p* < 0.05.

The mechanical properties of cells covering the construct were assessed with the AFM (Figure 4a). There was no significant difference among the Young’s modulus of the different samples. However, the current group tended to have lower values of the cell Young’s modulus (3.2 [2.8;6.7] kPa) compared with other groups (median 5–6 kPa). At the level of the whole mucosa-like model, the mechanical properties were measured by the microindentation technique (Figure 4b). These properties are mostly determined by the collagen membrane structure as the basis of the model. The initial collagen membrane without cell incubation was stiffer (19 [14;22] kPa) than other studied groups. Notably, mucosa-like models in the groups of NiCr–AgPd and NiCr–AgPd-i had higher Young’s moduli (14 [5;25] kPa and 11 [7;19] kPa, respectively) than other groups (4–7 kPa) with cell incubation, but not statistically significant.

## 4. Discussion

The study aimed to evaluate the different effects of oral galvanism on the keratinocytes that constitute the superficial layer of the oral mucosa [34]. We developed a cellular mucosa-like model based on collagen, which is the main component of the extracellular matrix in the living organism [35]. The line of keratinocytes (HaCaTs), on which studies of dental materials have been previously carried out [20,36,37], was chosen as the cellular component of the model since the properties of this cell line are stable, standardized, and reproducible [38]. HaCaTs were grown at the air–liquid interface since this method allows for maximal imitation of the epithelial natural growth environment [39,40,41,42].

Taking into account the relatively short timeframe of the current experiment (28 days compared with in vivo alloy chronic toxic effects lasting for years [43]), we decided to use the couples of dental alloys with the highest level of potential differences, Ti64–AgPd and NiCr–AgPd, as having potentially the highest risk of oral galvanism [44]. Moreover, the method of ion collection due to galvanic corrosion of chosen alloy couples turned out to be relevant because it was shown that the resistance of artificial saliva decreased after their incubation for 72 h.

In this work, we systemically assessed the effect of galvanic couple exposure (producing constant current and alloy ions simultaneously), the separate effect of alloy corrosion ions, and the separate effect of constant current on the developed mucosa-like model. Comparing the effects of galvanic couples (Ti64–AgPd and NiCr–AgPd) and their extracted ions, we showed a decrease in cell proliferation in all these groups. However, the cytotoxic effect was more pronounced in the case of galvanic couples rather than in the ion groups. This finding confirms the hypothesis of the synergetic damaging effect of galvanic couples due to generated current and metal ion release [9].

Apparently, for each galvanic couple, the dominant cytotoxic component may be different. In the Ti64–AgPd couple, cell damage was mainly associated with generated galvanic current since the ion group showed no statistical difference from the control group in terms of cell proliferation, while the galvanic couple group showed a statistically significant cytotoxic effect. At the same time, for the NiCr–AgPd groups, there was no statistically significant difference between the galvanic couple and ions. Nevertheless, both groups showed significantly decreased proliferative activity of cells compared with the control group. Therefore, the dominant damaging factor could not be clearly identified.

However, the contribution of Ni^2+^ ions to cytotoxicity was unequivocal. When comparing isolated exposure to corrosive Ti and Ni ions, the latter caused a statistically significant decrease in proliferative activity compared with the control group. Moreover, despite the fact that the Ti64–AgPd couple had a higher potential difference than the NiCr–AgPd couple, the latter pair turned out to be more toxic for cells: the NiCr–AgPd couple caused a slightly greater decrease in cell proliferation than the Ti64–AgPd couple. As well, greater toxicity of the NiCr–AgPd couple is detected according to a higher secretion of IL-6 that is produced by various cell types, including keratinocytes, and plays an important role in the inflammatory response [45], as well as the processes of oncogenesis and the development of oral squamous cell carcinoma [46]. The obtained results are consistent with the studies of other authors, in which Ni^2+^ ions also had a strong toxic effect on mucosal cells [47].

However, in the current experiment, the current group had the greatest damaging ability. Galvanic current of 10 µA was used for comparison because of the following reasons: (1) in the real oral environment, the galvanic current may increase significantly due to the activity of oral flora [48], the use of fluorine-based oral care products [49] or aggressive antiseptic solutions [50], and a decrease in salivary pH [51]; (2) the strength of the galvanic current in dental implant/suprastructure systems may reach the level of 12 µA [11], and in an in vivo study, the registered galvanic current in people with clinical manifestations of oral galvanism varied from 7 up to 25 µA [52].

The current of 10 μA reduced cell metabolic and proliferative activity by a multiple compared with other groups, as well as increased the expression of proinflammatory interleukin IL-6. Visualization of the samples using live/dead staining after current exposure also showed almost complete destruction of the cell layer on the surface of the mucosa-like model, while the areas with live cells were randomly scattered and corresponded, apparently, to the areas with the lowest current values. In addition, the current exposure apparently passed through the entire thickness of the collagen membrane since the cells also did not migrate deep into its thickness, which was observed for the other groups.

When studying the mechanical properties of the mucosa-like construct by AFM, no statistical differences in the elastic modulus (Young’s modulus) of the cell layer were found. As shown before, changes in the mechanical properties of the cell layer are related to their functional state and change during the cytotoxic actions [53]. Nevertheless, there was a tendency for Young’s modulus to decrease in the current group compared with the others, which also proves its strong toxic effect on cells.

The change in the macromechanical properties of collagen membranes on which the cells were cultured was interesting. The decrease in the elasticity of collagen membranes was probably related, to some degree, to structural degradation by the secreted cell ferments (e.g., MMP-1 [54]). In contrast, this was not as significant in the NiCr–AgPd galvanic couple and the ion groups; the observed effect could be caused by the ability of the Cr^3+^ ions to crosslink collagen molecules, thus hampering the process of its degradation [55,56].

Finally, the mechanism of cell death of the mucosa-like model cells by the current, as the most damaging factor we identified, appeared to be unrelated to caspase-3 activation. Indeed, the mechanism of cell death after current exposure may result from caspase-independent pathways of apoptosis activation [57,58] as well as necrosis due to membrane electroporation [59,60].

The present study has clinical significance since the issue of oral galvanism remains actual and arouses the interest of researchers and dental practitioners [9]. According to the obtained results, we may hypothesize that mouth burning and oral mucosa reactions are more likely associated with galvanic current than with an ion cytotoxic effect after the placement of metallic oral appliances. It was shown that even biocompatible and corrosion-resistant dental alloys might form a galvanic couple in the oral environment and cause damage to the oral mucosa with a low-strength constant current [2]. Interestingly, the studied galvanic couples did not cause serious cell damage, while the current alone did. It means that normally, the strength of galvanic current between dental alloys is too low to cause significant changes in oral mucosa, so people with galvanic couples may have no clinical signs of oral galvanism [61]. However, in some patients, the strength of galvanic current may reach the level of 25 µA, which may result in multiple side effects [52]: the risk of oral galvanism depends on many local and systemic factors, such as potential differences between dental alloys, saliva properties, dietary behaviors, and individual sensitivity of oral mucosa [26].

## 5. Study Limitations and Future Perspectives

In this study, we used an immortalized epithelial cell line from adult skin (HaCaT), which is not “native” to oral mucosa. In addition, it is worth noting that in the created mucosa model, the cells did not have multilayering. Probably, it was related to their migration inside the porous collagen matrix instead of forming multilayers, and therefore, we named the created model “mucosa-like”.

In future experiments, for better simulation of oral mucosa and greater extrapolability of the obtained data, it is worth developing full-thickness gingival equivalents with a more native structure. Besides keratinocytes, they also have a layer of fibroblasts, while these cells (immortalized or primary) are obtained directly from the soft tissues of the oral cavity [62,63].

This study was performed in a proof-of-concept style: we compared different toxic factors on a mucosa-like model for 28 days and showed that current exposure has a more damaging effect than ionic exposure. Nevertheless, it is not entirely dismissible that ions may cause similarly damaging effects on cells when exposed for longer periods.

Also, it should be taken into account that we used a relatively high-strength constant current and the most unfavorable combinations of dental alloys to achieve effects in a short period of time. In real clinical practice, more favorable combinations of alloys are commonly used, which result in lower galvanic currents. However, even the low-strength galvanic current may cause damage to the oral mucosa over a longer period of time.

Moreover, within this model, we had no opportunity to reproduce allergic reactions to metal ions, which may also play a role in the development of side effects associated with oral galvanism [8]. Therefore, further experiments with longer exposure times are required, as well as the use of additional research methods to detect possible “hidden” effects affecting other targets in the cells, influencing their proliferation, death, or transformation into cancer cells.

For this, the use of next-generation testing platforms like mucosa- or gingiva-on-a-chip [64,65] could be valuable. They will allow for better mimicking not only the native structure of the mucosa but also native (dynamic) conditions in the oral cavity: changes in pH, temperature, saliva renewal cycle, etc. This will undoubtedly allow for ensuring the impact of damaging factors within more physiological limits to study their impact at a qualitatively higher level.

## 6. Conclusions

Galvanic current is supposed to be the leading damaging factor in the cytotoxic processes associated with galvanic coupling of metallic intraoral appliances in the oral cavity, especially in the short-term period. This statement is supported by the fact that the renewal of saliva in the oral cavity occurs constantly, which additionally contributes to a better flow of current between the metals of different alloys on the one hand. On the other hand, it contributes to a faster washout of ions formed as a result of corrosion. According to this finding, we hypothesize that clinicians should pay more attention to providing preventive measures to minimize the risk of galvanic coupling in oral cavities and the possible electric current strength. The data related to potential differences between common dental alloys presented in our study may be useful for dental practitioners for materials selection and treatment planning.

However, this by no means cancels the contribution of ions released during corrosion to the synergistic toxicity of alloys to mucosal cells, and for some ions (e.g., Ni^2+^), the synergistic contribution may be extremely significant and, possibly, in the long term, no less harmful. Therefore, the cytotoxicity of dental alloys should be assessed comprehensively; no “ideal” uniform treatment plan exists, and decisions should be made in a personalized manner based on the specific galvanic couple. Dentists should take into account all possible risk factors for oral galvanism and pay special attention to conditions of oral mucosa when planning prosthetic rehabilitation.

## Data Availability

The data presented in this study are available in article.
